# BED Estimates of HIV Incidence: Resolving the Differences, Making Things Simpler

**DOI:** 10.1371/journal.pone.0029736

**Published:** 2012-01-03

**Authors:** John Hargrove, Cari van Schalkwyk, Hayden Eastwood

**Affiliations:** Department of Science and Technology, National Research Foundation, Centre of Excellence in Epidemiological Modelling and Analysis (SACEMA), University of Stellenbosch, Stellenbosch, South Africa; University of Sao Paulo, Brazil

## Abstract

**Objective:**

Develop a simple method for optimal estimation of HIV incidence using the BED capture enzyme immunoassay.

**Design:**

Use existing BED data to estimate mean recency duration, false recency rates and HIV incidence with reference to a fixed time period, *T*.

**Methods:**

Compare BED and cohort estimates of incidence referring to identical time frames. Generalize this approach to suggest a method for estimating HIV incidence from any cross-sectional survey.

**Results:**

Follow-up and BED analyses of the same, initially HIV negative, cases followed over the same set time period *T*, produce estimates of the same HIV incidence, permitting the estimation of the BED mean recency period for cases who have been HIV positive for less than *T*. Follow-up of HIV positive cases over *T*, similarly, provides estimates of the false-recent rate appropriate for *T*. Knowledge of these two parameters for a given population allows the estimation of HIV incidence during *T* by applying the BED method to samples from cross-sectional surveys. An algorithm is derived for providing these estimates, adjusted for the false-recent rate. The resulting estimator is identical to one derived independently using a more formal mathematical analysis. Adjustments improve the accuracy of HIV incidence estimates. Negative incidence estimates result from the use of inappropriate estimates of the false-recent rate and/or from sampling error, not from any error in the adjustment procedure.

**Conclusions:**

Referring all estimates of mean recency periods, false-recent rates and incidence estimates to a fixed period *T* simplifies estimation procedures and allows the development of a consistent method for producing adjusted estimates of HIV incidence of improved accuracy. Unadjusted BED estimates of incidence, based on life-time recency periods, would be both extremely difficult to produce and of doubtful value.

## Introduction

For infections of long duration, prevalence estimates are less informative than incidence as measures of the state and trajectory of an epidemic. For HIV, where infection durations can exceed a decade even for patients not on antiretroviral therapy (ART) – and can be even longer for patients who are – incidence estimates are particularly important. However, whereas HIV prevalence is relatively easy to measure, HIV incidence is much more difficult. Even the so-called “gold standard” approach, involving follow-up of cohorts of initially HIV negative cases, is not without bias and is costly, time consuming and logistically challenging.

Ideally one could calculate incidence from the samples collected in cross-sectional surveys used to estimate HIV prevalence – if it were possible to identify, from among the HIV positive cases, those that had become infected within some specified period prior to the time of the survey. Various methods have been suggested for identifying so-called “recent infections”; none is so far entirely satisfactory and research into improved methods is on-going. A widely used approach is the BED Capture Enzyme Immuno-Assay (BED-CEIA or simply BED) assay which has been used alone, or in combination with an avidity assay, to estimate HIV incidence [1].

A common problem with the assay is that, when applied to arbitrary cross-sectional survey data, the resulting HIV incidence often over-estimates the true values [2]. This problem has led to considerable discussion [3], [4], [5], [6], [7], [8], [9], but to no general agreement on how best to proceed. In this paper we suggest a fresh approach, which resolves difficulties with the BED method and, more generally, provides a simple improved approach to HIV incidence estimation using biomarkers.

## Methods

The BED method is based on the increasing proportion of anti-HIV-1 immuno gamma globulin (IgG) in total IgG following seroconversion [1]. People are classified as ‘recent’ seroconverters if their blood samples test positive by a standard HIV-1 enzyme-linked immunosorbent assay and have a normalized optical density (OD) below a pre-set cut-off (*C*) on the BED assay.

**Figure 1:**
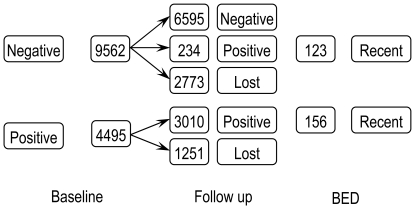
Summary of baseline and 12-month postpartum HIV results and, for the latter, of the BED results for HIV positive cases.

The method was first characterized for use on clade-C HIV virus using samples collected during the ZVITAMBO Trial, carried out in Harare, Zimbabwe between October 1997 and January 2000 [3]. Of 14,110 women and their babies, recruited within 96 hours postpartum, 9562, 4495 and 53 mothers tested HIV negative, positive and indeterminate, respectively (Figure 1). All were followed up at 6-weeks, and at 3, 6, 9 and 12-months postpartum; surviving mothers were retested for HIV at each follow-up visit. Of the original HIV negative mothers *N* = 6595 still tested negative at 12-months postpartum, at which time all HIV positive mothers were also tested using BED. Of the *P_N_* = 234 seroconverters, and the *P_P_* = 3010 who had previously tested positive at baseline, *R_N_* = 123 and *R_P_* = 156, respectively, had a BED OD<0.8 when they were tested at 12-months postpartum. These *R* = *R_N_* +*R_P_* cases were classified as *recent* seroconversions – where “recent” means that seroconversion is supposed to have occurred within the previous 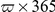 days – this period is termed the *mean recency duration* (previously “window” [3]), appropriate for the chosen BED cut-off −0.8 in the case of the ZVITAMBO study.

## Results

### Defining the problem

The detailed follow-up data allowed the estimation of HIV incidence over 12 months directly from the frequency of seroconversions observed at each of the first five post-natal visits, and also allowed the estimation of ϖ and, thereby, the BED incidence. If seroconversions are uniformly distributed during the first 12-months postpartum their number is estimated by *R*/ϖ and the *cumulative incidence*, or *risk* (*J*_0_) that an HIV-1-negative woman seroconverted in the 12-months postpartum, can be estimated by:
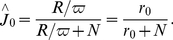
(1)where *r*_0_ = *R*/ϖ, *N* is the number testing HIV negative at follow up, and where we use the symbol *J* to indicate annualized *risk* of infection, reserving the symbol *I* for the incidence *rate*, see below.

With *N* = 6595, *P* = *P_N_* + *P_P_* = 3244, *R* = 279 and ϖ = 0.512 years (187 days), the unadjusted BED incidence estimate is 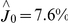
(95% CI; 6.7%–8.5%). By comparison the follow-up estimate was 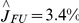
(95% CI, 3.0–3.8) [3], 55% lower than the BED estimate: this discrepancy needs to be explained.

One suggestion is that the difference is primarily due to the existence of a proportion (ε) of the population that tests as recently infected despite having been HIV positive for very much longer than ϖ – typically more than twice as long. For example, in the ZVITAMBO study, 156/3010 = 5.2% HIV positive cases tested recent by BED at 12-months postpartum, despite having been infected for more than a year. Various mathematical adjustments have been suggested to counter this problem [3], [4], [10].

An alternative interpretation is that the problem arises because ϖ, as estimated for instance in [3], under-estimates the population mean recency period. This period, henceforth ϖ*_P_*, is defined as the total time that an HIV case tests as a recent infection during his/her entire life after infection – including times when the case re-enters the recent state. It has been argued that, if ϖ*_P_* were appropriately estimated, adjustment would largely be unnecessary [5]. Moreover, it was claimed [5] that two suggested adjustment procedures either had no effect on the incidence estimates [4], or contained a mathematical error, which led to substantial under-estimates of HIV incidence [3].

This view has been countered with the demonstration that, for there to be a match between follow-up and BED unadjusted estimates of incidence, an unfeasibly long value of ϖ = 1.202 years (439 days) would be required [6]. This argument was, in turn, rejected on the grounds that there is no reason to suppose that the two incidence estimates *should* be the same [7]. The reason for this is that the follow-up estimate refers only to the 12-months postpartum period, whereas any biomarker estimate applied at 12-months postpartum will be estimating the incidence averaged over a longer period – including, in the ZVITAMBO example, the pre-partum (pregnancy) period when incidence will likely be higher.

### BED and follow-up incidence estimates over the same time period

This last argument, which may be regarded as the point of departure for the present paper, refers to the situation where the BED analysis is applied to all of the ZVITAMBO 12-month data. Consider, however, the case where the BED analysis is applied to the 12-month data that arose only from the 9562 women who tested negative at baseline – *i.e.*, the 6595 who were seen at the 12-month follow-up visit and who still tested HIV negative at that time, and the 123 cases among the seroconverters who tested recent by BED (Figure 1). Now both the BED and follow-up incidence estimates refer, unequivocally, only to the 12-months postpartum period – because, given our data selection, no case has been HIV positive for longer than 12 months. The BED analysis applied to these data should provide approximately the same incidence as the follow-up procedure, and the appropriate value for the mean recency period in Equation (1) is thus the value that ensures this equality. Setting *N* = 6595, *R* = 123 and 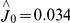 in Equation (1):
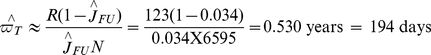
where ϖ*_T_*, is now defined as *the mean time spent in the test-recent state while infected for less than T* [11], where *T* = 365 days for the ZVITAMBO analysis. We return later to the problem of how best to estimate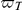; but notice that the value of 194 days is only 3.7% higher than the 187 days estimated from the pattern of increase of BED OD among seroconverters in the ZVITAMBO study [3].

Notice also that ϖ*_T_* will indeed under-estimate ϖ*_P_*, as correctly pointed out in [5]; and whereas ϖ*_T_* is clearly the appropriate mean recency period for the problem analyzed in the previous paragraph the question arises whether we should be using ϖ*_T_* or ϖ*_P_* when analyzing normal cross-sectional survey data.

### Reducing the problem

This question can be addressed by the following thought experiment. Suppose we were presented with the full 12-month ZVITAMBO data as shown in Figure 1 but where everybody was being seen for the first time – *i.e.* we simply saw 6595 HIV negative and 3244 positive cases. Suppose now that we had a test that was similar to the currently available BED test, except that it *always* correctly identified positive infections as long-term – as long as they had been HIV positive for more than one year. Under such circumstances we would only see 123 BED-recent cases, we would ignore all other HIV positive cases and the analysis would be reduced to the above situation involving these 123 recent cases and the 6595 negatives. We would then, again, be estimating an incidence that referred only to the previous 12-month period and it would again be appropriate to use ϖ*_T_* as the mean recency period. One could equally reduce the analysis to this simpler situation if clinical information allowed the identification of individuals HIV infected for more than a year – regardless of their BED test result.

### Extension to all BED incidence estimates

The simple situations described in the previous section do not generally exist in African settings where people in cross-sectional surveys are subject to anonymous testing, and where there is no information about previous HIV testing history. The question then arises: “Is there, nonetheless, any way in which we can reduce the BED analysis to the simpler situation involving only HIV negative cases and the number of cases that have been HIV positive for less than some defined convenient short period of time, *T*?”

The answer to this question is “yes” – as long as we have a good estimate of ε, the *proportion* of people in the population under study that will test recent by BED if they have been HIV positive for time > *T*. Applying ε to the BED results of a cross-sectional survey we can estimate the *number* of these long-term false-recent cases *without needing to know which those cases are*. We then simply subtract this number from *R* (the total number testing recent – correctly or falsely) and the analysis reverts to that involving only the HIV negative cases – and the remainder of the recent cases. The incidence over the time period *T* prior to the survey is then estimated as before from Equation (1), using the mean recency ϖ*_T_*, where ϖ*_T_* is expressed as a proportion of *T*. The following provides an outline (see Supporting Information S1 for derivation) of the algorithm required to achieve this.

If *R* provides a first estimate of the number of recent seroconversions then, assuming a uniform distribution of seroconverters over the previous time *T*, a first approximation of the estimated number (*r*_0_) of cases that have seroconverted in the previous time *T* is given by *r*_0_ = *R/*ϖ*_T_* , and a first estimate of the incidence is, using Equation (1), 
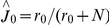
. The first estimates (*p*_1_) of the number who have been HIV positive for longer than *T* is 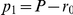. But, by assumption, a proportion ε of the *p*_1_ cases, that have been HIV positive for longer than *T*, still test recent by BED. A better estimate of the number of cases who seroconverted in time *T* prior to the survey is thus given by 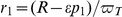, which provides an improved estimate, 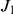, of the incidence and the basis for an iterative procedure, which converges rapidly. With the ZVITAMBO input data, and assumed values of ϖ*_T_* = 0.513 (187 days) and ε = 0.0517, as *i*→∞, *r*_i_→123, ε*p*_i_→156, the number known to be long-term false-recent cases (Figure 1), and 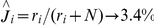, the value observed from the follow-up study.

The exact match to the follow-up incidence in the ZVITAMBO Trial is artificial, because we have used the value of ε derived from the observed number of long-term false-recent cases in the same data set. As pointed out previously, any adjustment procedure will only be useful if estimates of ε are applicable to the analysis of BED data from other similar populations. The present example is merely provided to establish the principle of how the adjustment procedure functions. The idea is simply to reduce the problem to the equivalent situation where we estimate incidence from the number of recent seroconverters among cases that were HIV negative at time *T* prior to the survey. It is then appropriate to use ϖ*_T_* as the estimate of the mean recency period and the incidence refers to the period, *T*, over which both ϖ*_T_* and ε are defined. Nor does the period over which the incidence is estimated need to be 12 months: this period was used in the analysis of the ZVITAMBO data only because clients were followed most intensively at the 12-month visit and thus provided the most complete data for BED analysis.

### Closed form solution

Formally, the above iterative procedure converges to the following closed form solution for the adjusted risk of infection:
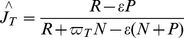
(2)where ϖ*_T_* is a (dimensionless) proportion of *T*. For comparison with more recent results [10], note that the same derivation procedure (see Supporting Information S1) also produces an estimator for the instantaneous incidence *rate*:
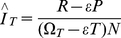
(3)where 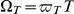. A more formal mathematical development produces the same result [11].

Whereas Equation (2) was originally presented as a simplification of the adjustment in [4], it was actually derived as shown in the Supporting Information S1. This shortcut, and the failure to note the importance of strictly relating the mean recency period to those cases that had been HIV positive for less than a year, has led to considerable misunderstanding, which is resolved in the following sections. Note that whereas, in making the simplification (see equation (3) in [3]) we set ϖ = 0.5, the derivation here (see Supporting Information S1) shows that this assumption is not necessary; nor is it necessary to stipulate that the incidence is to be estimated over a year (see above) and finally, it is not necessary to assume equality between the sensitivity (σ: the probability that a case HIV positive for time *t*<ϖ*_T_* has BED OD<*C*) and short-term specificity (ρ_1_: the probability that a case HIV positive for time ϖ*_T_*<*t*<*T* has BED OD≥*C* [3].

### Definitions of the long-term false-recency rate

Equation (2) has been criticized on the grounds that, under the assumptions of its derivation, ε must necessarily take the value zero [5]. The problem lies in the use of different definitions of the mean recency period and the long-term false recency rate. The symbol ε was first defined in this context from the ZVITAMBO data as the proportion of cases that were “misclassified as recent seroconverters at least a year after they first tested HIV-1 positive” [3]. In presenting Equation (2), however, ε was also equated with 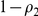 as used in [4] and the problem is that these workers effectively use two estimates of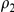 which differ in a crucial way. The value actually used in their adjustment was “the overall rate of false positives (1-specificity)” and was “based on analysis of specimens from longer-term-infected individuals not known to have clinical AIDS, opportunistic infections, or to be on treatment”. This practical definition is the same as the one used for ε in [3] and makes no assumption regarding the source of the false positives.

However, in the analysis of the probability of remaining in the recency period,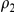 was also defined as the specificity over the period greater than twice the recency period after seroconversion, “where the curve is flat” [4]. This is the sense in which Brookmeyer [5], [9], and also McWalter and Welte [10], [12], use the term and is the probability, *P_NP_* [10], that a case never leaves the recent state.

A necessary condition for these two definitions to be congruent, with *P_NP_* = ε, is that no cases exist where persons re-enter the recency state after having exited. This is explicitly assumed in [5] but evidence to the contrary had already been published [3]. The point will now be explored more fully since it lies at the heart of the confusion and its clarification should lead to a reconciliation of the divergent views on the best way to estimate HIV incidence from BED data.

### Reversions to the recent state: time courses of changes in BED optical density

**Figure 2:**
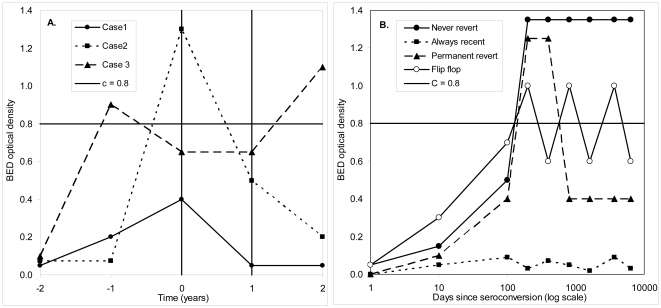
A. Patterns of changes in BED optical density showing the range of scenarios actually observed in the ZVITAMBO Trial and possible changes before and after the observational period. Clients were first seen at time 0 and then again a year later. For purposes of illustration it is assumed that all cases had seroconverted two years previously at which time they had a BED OD close to zero. See the text for discussion of possible changes in BED OD before the clients were tested at *t* = 0 and again after they were tested at *t* = 1. B. Possible long-term changes in BED OD following HIV infection.

In Figure 2A we sketch the combinations of OD values observed at baseline (*t* = 0) and 12-months later (*t* = 1) in ZVITAMBO – and then consider possible values of the OD for times *t*<0 and *t*>1 that are consistent with these observations. Of those who were HIV positive at *t* = 0, and tested positive again at *t* = 1, 95% (2607/2749) had a BED OD>*C* = 0.8 at *t* = 1; these “normal” scenarios are not illustrated in Figure 2. The concerns surround the histories, observed and implied, of the 142 cases illustrated by Cases 1–3, which were HIV positive at *t* = 0 but then had a BED OD<*C* at *t* = 1. Case 1 typifies the situation, observed in 103/142 (73%) cases in ZVITAMBO, with BED OD<*C* both at *t* = 0 and *t* = 1. Such cases, if they had an OD<*C* for all *t*>0, constitute the group sometimes used to define ε [5], [9] and, equivalently, *P_NP_* [10].

But this is not the way that ε was defined in [3], because the above definitions exclude the situations typified by Case 2, and explicitly reported for 39 cases in ZVITAMBO, ([3]; page 515, first paragraph), where OD>*C* at *t* = 0 and OD<*C* at *t* = 1. That is to say, 39/142 = 27% of the cases constituting the original definition of ε were BED long-term at baseline but had reverted to the recent state a year later. Moreover, 27% is only a lower bound for the probability of reverting. Thus, since cases *can* revert, we must allow the possibility that any particular case with OD<*C* at *t* = 0 and *t* = 1 had actually both left the recent state, and then reverted, at some time prior to *t* = 0, as illustrated in Case 3. In principle, therefore, we can only say from the ZVITAMBO data that the probability of reverting lies between 27% and 100%. Possible time courses of changes in BED OD over the life of an HIV positive case are summarized in Figure 2B.

Indications that reverting cases form an important proportion of ε were obtained from the ZVITAMBO seroconverting panels for cases followed up for sufficiently long that they were *known* to have been HIV positive for 365 days – *i.e.* the time between the first HIV *positive* test and some later test exceeded 365 days. There were 51 such seroconverting cases, only one of which (2%) never left the recent state. This provides a first estimate of *P_NP_*, which is less than half of the estimate of ε. We caution, however, that even though the one case that failed to leave the recency period was followed for >700 days after seroconversion, there is no guarantee that the OD failed thereafter to exceed 0.8. In this sense 2% is an upper bound for *P_NP_*. On the other hand, this is a very crude estimate given the small sample size; the 95% confidence interval for the estimate ranges from 0.1% to 10.5%. Further information on this area is now of extreme importance.

### Implications of reversion to the recent state for models of BED

**Figure 3:**
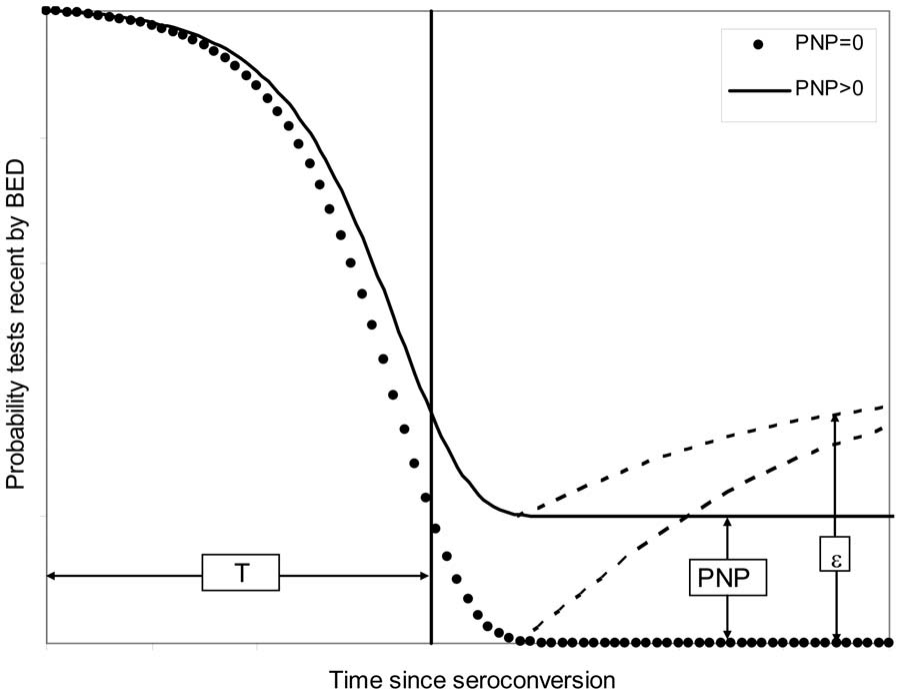
Changes in the probability of testing recent by BED with time since HIV infection. For PNP = 0 there are no “non-progressors” – *i.e.* it is assumed that every person, at some point, has a BED OD>*C*, the pre-set OD cut-off. For PNP>0 some people never have an OD>*C*. In both cases, however, it is allowed that some cases may revert, temporarily or permanently – *i.e.* that the OD declines from >0.8 to <0.8. Note that this implies that the level of ε may vary with the time since seroconversion and, in particular, may not even (as illustrated for simplicity) increase monotonically with time.

Models for the BED method have assumed a situation, pictured in Figure 3, where the probability of a case remaining in the recent state declines monotonically, either to some low constant level >0, termed *P_NP_* in [10], or ultimately to zero if, as argued in [5], no case stays in the recent state for more than about three years. In either event the function is used to derive an identity relating the BED sensitivity (σ) and the short and long term specificities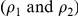:
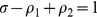
(4)If this identity is correct and if, as assumed in [3], 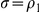, then it follows that 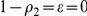, contrary to the assumption in [3] and pointing therefore to a mathematical error in Equation (2) [5].

There are two problems with the argument: 1) It is based on a population estimate of the mean recency (ϖ***_P_***), as opposed to the estimate actually used to estimate ε, which was approximately the same as ϖ***_T_*** and thus << ϖ***_P_***. 2) It failed to take into account published evidence that cases can revert to the recent state at time(s) *t*>*T* , so that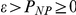. The argument in [5] thus provides no evidence of a mathematical error in Equation (2). Indications, from a numerical example [5], that Equation (2) massively under-estimates HIV incidence, leading inevitably to negative estimates of HIV incidence where there are large numbers of prevalent long-standing infections, arise from confusion over the definitions of ϖ***_T_*** *vs* ϖ***_P_*** and ε *vs P_NP_*_._

This point is illustrated by a numerical example using the ZVITAMBO data. Suppose the data were as shown in Figure 1, except that 100,000 women tested HIV positive at baseline and again 12 months later. Then at 12-months postpartum the data would have consisted of *N* = 6595, *P* = 234+100,000 and *R* = 123 + ε ×100,000≈5289 – assuming 5.166% of all women HIV positive for more than 12 months test recent by BED. Inserting these data into Equation (2) produces a value of *J_T_* = 3.5% as with the original ZVITAMBO data. Negative estimates of incidence do not thus arise from Equation (2) simply through large numbers of prevalent long-standing infections. They can, however, arise from the use of inappropriate values of ε [3], and/or the mean recency period and/or from counting errors.

Given the reality of cases reverting to the recent state, what is evident from Figure 3 is that functions presented as relating the time since infection to the probability of being in the recent state [5], [10], do not decline monotonically and are thus not survival function in the normally understood sense. During the period [0, *T*] the BED OD generally increases rapidly and the vast majority of cases will therefore leave the recent state at some time *t*<*T*. Thereafter, however, if cases start reverting to the recent state, the “survival” function increases – in some way that we have not yet measured or understood – from *P_NP_* towards some higher level such that if we take a cross-sectional survey we see a proportion ε>*P_NP_* of cases which test recent by BED when they are known to have been HIV positive for *t*>*T*. Models for BED [5], [10], need to be modified to take account of cases that, permanently or temporarily, re-enter the recent state. An improved model of this type has now been developed [11].

### Estimating ϖ_P_ and ϖ_T_

We now return to the problem of estimating mean recency periods from follow-up data. With reference to the scenarios encapsulated in Figure 2A and B, the ideal case would be one where, invariably, the OD increases with time, eventually leaving the recent state (OD<*C*) not to return – until perhaps the patient develops late stage AIDS or initiates ART. It is assumed that such patients could be identified and excluded from the recency estimation procedure. The mean recency is then relatively easily estimated since one is only looking for the first passage time to *C*. The ZVITAMBO Trial produced, however, at least one case where follow-up for at least 714 days post-seroconversion never produced a BED OD>0.1. One must allow the possibility that this case would stay in the recent state indefinitely. Similarly, some cases do revert to a recent state and we cannot know whether those that revert stay in the recent state indefinitely or whether they “flip-flop” one or more times between the states.

These complications suggest that ϖ*_P_*, as defined in [5], could never practically be estimated. Thus, it is stated that the mean recency period should include the total time for which OD<*C* – *i.e.*, the initial time plus any subsequent times when the OD dips below *C*. But if the OD might drop below *C* at any time, or shows no signs of ever being greater than *C*, or flip-flops between the states, then this means that in order to estimate ϖ*_P_* it would be necessary to follow individual seroconverters for their entire lives. This was never feasible, and will now be quite impossible given the ethical objections to following HIV positive persons for the requisite time without giving them access to ART. Given these problems and that the full distribution of recency periods has a very long tail it is also obvious that estimates of ϖ*_P_*, even if they could be obtained, would come with unacceptably large errors.

The motivation for estimating the population mean recency period ϖ*_P_* is to obviate the adjustment procedure [5]. Even if this were possible, which seems unlikely given the foregoing, it may not even be desirable. Thus, as pointed out in [5], the cross-sectional biomarker approach, applied using ϖ*_P_*, estimates a time-weighted average of incidence prior to the time of the survey, where the weighting function is the backward recurrence time density which depends on the entire mean recency period distribution including its tail. In the ZVITAMBO context, the BED method would then estimate incidence over a period that could extend, for some unknown time, into the pre-partum (pregnancy and even pre-pregnancy) period. Having an incidence estimate which spans some ill-defined time period, and is dependent on some complex and currently undefined weighting function does not seem particularly helpful.

By contrast, as evidenced by the ZVITAMBO study, the estimation of a censored mean recency period, ϖ*_T_*, and the associated adjustment factor ε, is a relatively straightforward matter and the estimates come with acceptable levels of error. Moreover, the procedure gives rise to incidence estimates that refer to a well defined period, *T*, of order one year. Further major advantages are: i) One can largely ignore the effects of mortality, which should be minimal during periods of order one year post-seroconversion. ii) There is no need to address the difficult matter of weighting in situations where we can assume incidence has changed little over time *T*.

In estimating the mean recency period from the ZVITAMBO BED data, seroconverter panels were excluded if the range of ODs did not span the pre-set OD cut-off (*C*) [3], [13]. Panels were thus excluded if the OD at the time of the first HIV positive test was greater than *C*, or if the maximum OD among all BED tests was less than *C*. The net effect of this was to produce an approximation to a censored mean recency period, ϖ*_T_*, as defined above. An unbiased estimate of ϖ*_T_* is provided by *R/S* [11] where *S* is the number of HIV positive cases observed at time *T*, among those HIV negative at time 0, and *R* is the number of these seroconverters that test recent by BED. For ZVITAMBO, with *T* = 365 days, 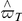 = *R/S* = 123/234 = 0.526 years = 192 days (Figure 1). The estimating procedure for ϖ*_T_* in [3] thus produced a biased result – but the difference (187 days *vs* 192 days) is small, as are the differences between these two estimates and the 194 days required to produce an exact match between the ZVITAMBO BED and follow-up estimates of HIV incidence (see above). Given the similarity in the results derived from these various approaches to estimating ϖ*_T_* it seems likely that further improvements in its estimation will lead only to minor improvements in the accuracy of the BED method of estimating HIV incidence.

### Biases in BED adjustment procedures

A more complete, and complex, development of the theory underlying the use of biomarker estimates of HIV incidence provided, *inter alia*, a maximum likelihood estimate of the adjusted BED incidence *rate*:
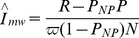
(4)where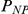, as defined above, is the probability that the OD never exceeds the pre-set OD cut-off *C*. This formulation was used to estimate the biases in the adjustment given by Equation (2) [12]. In practice the differences between the various adjustments are small compared with the counting errors. Thus, using the 12-month ZVITAMBO data, Equation (4) gives an annual incidence rate of 3.46%, compared to 3.65% from Equation (3). Moreover, Equation (4) has now been superseded and the new incidence estimator is identical to Equation (3), if both use the same mean recency period [11].

## Discussion

A recent review of measuring the HIV/AIDS epidemic repeats the idea that long-term-false-recent cases may be ascribed variously to assay non-progressors, elite controllers, persons in late-stage HIV and/or cases on ART [14]. The higher level of total IgG in African than in Western countries, due to higher exposure to other pathogens, also increases the risk of misclassification. While these cases must of course be dealt with individually, our analysis shows that even when they have all been removed, HIV incidence will still be over-estimated where there are cases that revert to the recent state. And that problem can, currently, only be dealt with using mathematical adjustments [3], [4], [12].

The importance of mathematical adjustments of BED estimates of HIV incidence arises from the fact that we cannot, even in principle, estimate the population mean recency period (ϖ*_P_*). Because we are unable to follow up cases for long enough, all estimates from follow-up data under-estimate ϖ*_P_*, and thus over-estimate HIV incidence. Restricting our view to a time period *T* means that we neither need, nor want, to estimate ϖ*_P_*. Instead we use ϖ*_T_* – which is easier to estimate – and then adjust for the number of cases that have been HIV positive for longer than *T*, and still test recent by BED.

The adjustments provided in Equations (2) and (3) will all only be as good as the value of ε – and therein lies a problem. If an important component of ε is due to reversion to the recent state, and if this reversion can occur at any time and is not solely or even mainly associated with late-stage disease, then we expect that ε might vary quite rapidly with time, even in a single population.

As pointed out in the original analysis of the ZVITAMBO data, high variability in ε would be sufficient to render the BED method of little general use [3]. The scientific community has been slow in investigating such variability and it is fruitless to speculate further about such variability and its origins. What is nonetheless clear is that there is an urgent need for tests that are able to identify long-term infections with much greater certainty than the BED method. Use of such a test, either by itself or in conjunction with the BED method, would constitute a major advance in estimation and would, inter alia, remove the necessity for mathematical adjustments in the estimating procedure.

## Supporting Information

Supporting Information S1 (DOC)
