# Supplementary material for: BED Estimates of HIV Incidence: Resolving the Differences, Making Things Simpler
Source: PLoS One. 2012 Jan 3;7(1):e29736. doi: 10.1371/journal.pone.0029736 (PMC3250478; doi:10.1371/journal.pone.0029736)
Supplement: Supporting Information S1 — (DOC) [file pone.0029736.s001.doc]

**Supporting Information**

***Adjustment for clients who test “recent” by BED but have actually been HIV positive for longer than a prescribed cut-off time T***

The uncorrected estimate of the risk of becoming infected with HIV over time *T* is given (see main text) by:

(1)

Where *N* is the number testing HIV negative, *R* the number, among *P* HIV positive cases, that also test recent by BED, *r_0_* = *R/ϖ_T_* is a first estimate of the number who have seroconverted in the previous time *T*, and *ϖ_T_* is a (dimensionless) proportion of *T*.

But seroconverters can be separated into two mutually exclusive sets: i) those who seroconverted in the previous time *T*; ii) those who have been HIV positive for > *T*. A first estimate$p_{1}$, of the number who have been HIV positive for time > *T* is thus given by:

$p_{1}=P-r_{0}$ (2)

By assumption, a proportion ε of the *p*_1_ cases that have been HIV positive for longer than *T* still test recent by BED at time *T*. A better estimate of the number of cases who seroconverted in time *T* prior to the survey is thus given by:

$r_{1}=\frac{1}{\varpi_{T}}(R-\varepsilon p_{1})$ (3)

and this, in turn, gives an improved estimate ($p_{2}$) of the number who have been HIV positive for longer than time *T*. Given the above fixed parameters *P*, *N*, *R*, *ε* and *ϖ_T_*, define the pairs of sequences (*p_i_*), (*r_i_*) for *i* = 1, 2 … by $p_{i}=P-r_{i-1}$ and $r_{i}=\frac{1}{\varpi_{T}}(R-\varepsilon p_{i})$ with$p_{0}=P$ and $r_{0}=\frac{R}{\varpi_{T}}$. Successive estimates of the incidence are thus given by *J_T_*(*_i_*) (*i* = 1, 2, 3 …) where $J_{T(i)}=\frac{r_{i}}{r_{i}+N}$

Since $J_{T(i)}=1-\frac{N}{r_{i}+N}$, the sequence (*J_T_*(*_i_*)) converges if (*r_i_*) does. Now, for any *k* = 1, 2 …

$$r_{k}=\frac{1}{\varpi_{T}}(R-\varepsilon p_{k})=\frac{1}{\varpi_{T}}(R-\varepsilon(P-r_{k-1}))=\frac{1}{\varpi_{T}}(R-\varepsilon P)+(\frac{\varepsilon}{\varpi_{T}})r_{k-1}$$

Write$\beta=\frac{\varepsilon}{\varpi_{T}}$. Consider (3) for *k* and (*k*-1) and subtract to obtain, with$\Delta r_{i}=r_{i}-r_{i-1}$:

$$\Delta r_{k}=\beta\Delta r_{k-1}=\beta^{2}\Delta r_{k-2}...=\beta^{k-1}\Delta r_{1}=\beta^{k-1}(r_{1}-r_{0})$$

So, for any *k* = 1, 2, ….

$$r_{k}-r_{0}=(r_{1}-r_{0})\sum_{j=1}^{k} \beta^{j-1}=(r_{1}-r_{0})\sum_{j=0}^{k-1} \beta^{j}=(r_{1}-r_{0})\frac{1-\beta^{k}}{1-\beta}$$

Thus $\begin{matrix} r_{\infty}=\lim_{k\to\infty} r_{k}=r_{0}+(r_{1}-r_{0})\left(\frac{1}{1-\beta}\right)=\left(\frac{1}{1-\beta}\right)(r_{1}-\beta r_{0}) \\ =\left(\frac{\varpi_{T}}{\varpi_{T}-\varepsilon}\right)\left(\frac{1}{\varpi_{T}}\left[R-\varepsilon p_{1}\right]-\left(\frac{\varepsilon}{\varpi_{T}}\right)r_{0}\right) \\ =\left(\frac{\varpi_{T}}{\varpi_{T}-\varepsilon}\right)\left(\frac{1}{\varpi_{T}}\left[R-\varepsilon\left\langle P-\frac{R}{\varpi_{T}}\right\rangle\right]-\left(\frac{\varepsilon}{\varpi_{T}}\right)r_{0}\right) \\ =\left(\frac{\varpi_{T}}{\varpi_{T}-\varepsilon}\right)\left(\frac{1}{\varpi_{T}}\left[R-\varepsilon P\right]+\frac{\varepsilon R}{\varpi_{T}^{2}}-\left(\frac{\varepsilon R}{\varpi_{T}^{2}}\right)\right) \\ =\frac{R-\varepsilon P}{\varpi_{T}-\varepsilon} \end{matrix}$

and

As noted above, is the adjusted *risk* of infection over time *T*. Authors often prefer to estimate incidence as an averaged *rate* (*I*) over time *T*, and best estimated for the situation where ε = 0 by:

When ε > 0 we replace *R* with $\varpi_{T}r_{\infty}$to get:

where $\Omega_{T}^{'}=\varpi_{T}T$ and $\Omega_{T}^{'}$ now has the dimension of time.
